# Exploring navigation of gender in a sample of clinically referred young people attending the gender identity development service

**DOI:** 10.1186/s13034-023-00627-6

**Published:** 2023-06-30

**Authors:** Daisy Haywood, María de Andrés, Una Masic, Polly Carmichael

**Affiliations:** Gender Identity Development Service, Tavistock and Portman NHS Foundation Trust, 120 Belsize Lane, London, NW3 5BA UK

**Keywords:** Gender-diverse, Social transition, Minority stress, Social connections, Mood, Transphobic bullying

## Abstract

**Background:**

Gender-diverse young people experience a cisnormative world and are subject to unique minority stressors, which have been found to contribute to adverse mental health. This research aims to understand the social and personal context unique to gender-diverse people that young people navigate prior to attending specialised services.

**Methods:**

The baseline measure of a newly developed questionnaire, the GIDS Gender Questionnaire (GIDS-GQ), was sent to all young people (or caregivers for those aged under 12) attending the Gender Identity Development Service (GIDS). Eighty-four young people and caregivers completed the questionnaire, with eighty-one included in the final sample (*M* = 15.77 years, *SD* = 1.83, range = 9–17; assigned female at birth = 72, assigned male at birth = 9). Questionnaires were emailed to participants via an online survey between one and three appointments with the Service. Data were collected between April 2021 and February 2022.

**Results:**

All young people had initiated a social transition, with 75.3% categorised as fully socially transitioned. More young people reported experiencing transphobic bullying (64.2%) and a lack of acceptance of their gender identity (85.1%) in the past (lifetime) than in the 6 months prior to attending the service (transphobic bullying: 12.3%; non-acceptance: 49.4%). 94.5% of the sample reported disliked body parts, most commonly breasts (80.8%), genitals (37%), and hips (31.5%). Participants most commonly reported a decrease in their mood (61.25%) and most areas of social connectedness.

**Conclusions:**

The majority of this sample had socially transitioned, were supported in their identification, and had experienced less transphobic bullying and non-acceptance prior to commencing services. However, young people continued to dislike their bodies, and experience low mood and social connectedness. Future research is needed to understand how clinical support can help reduce the impact of these external/distal minority stressors by promoting social connectedness, incorporating such learnings into clinical practice and subsequent policy in clinical work with gender-diverse young people.

## Background

“Gender-diverse” is an umbrella term used to describe a wide range of gender identifications outside of conventional gender norms [1]. Over the last decade, the number of young people referred to specialist services identifying as gender-diverse has increased significantly [2–4]. For some young people, the incongruence between sex assigned at birth and identified gender is highly distressing [5], and referrals seeking specialist care have increased globally [6–8].

Much of this distress has been attributed to the development of primary and secondary sex characteristics of the sex assigned at birth during adolescence [9–11]. In addition to these unwanted bodily changes, young people must also navigate a cisnormative world. The Gender Minority Stress Framework proposes that gender-diverse individuals experience internal/proximal stressors such as internalised transphobia and stigma, which occur after experiencing external/distal stressors such as discrimination, bullying, and/or non-acceptance [12–14]. Previous research with gender-diverse adults and young people suggests that these stressors may lead to increased risks of mental health difficulties, particularly if experienced consistently [15–17]. Bullying is an external stressor commonly reported by gender-diverse young people [18–21] but research is scarce on whether this bullying is transphobic in nature.

Social transition is often the first step a gender-diverse person takes to live as the gender that they identify with [22]. Typically, this may involve choosing preferred pronouns, name, and/or aspects of appearance to reflect one’s experienced gender identity [23]. This also includes considering with whom, where, and in which situations they may feel comfortable to present as their identified gender. Research indicates that each year an increasing number of clinically referred young people have socially transitioned before they are first seen in gender services [23–25]. This may be owing to long waiting lists to attend services resulting in young people socially transitioning during this wait.

Despite rates of social transition increasing, many gender-diverse young people are not treated in a way that reflects their gender identity. Misgendering (an incorrect assumption of one’s gender identity and/or use of incorrect pronouns), and use of birth-assigned rather than chosen name, are common and reoccurring external stressors for many gender-diverse people [14, 26]. Research suggests that misgendering and incorrect name use can have negative implications for the mental health outcomes of gender-diverse adults and young people [14, 27–29]. In parallel, acceptance of gender identity, such as others’ use of a chosen name, has been associated with improved mental health outcomes in gender-diverse young people [30].

The Minority Stress Model highlights social connectedness as a coping mechanism against minority stressors [12–14]. Previous research has aggregated existing measures to determine the quality of peer relations (the Peer Relation Scale; PRS), with findings suggesting clinically referred gender-diverse young people to have poorer peer relations than their cisgender siblings [31]. Cross-clinic comparison studies have also found a substantial relationship between negative PRS scores and behavioural and emotional difficulties for young people attending gender services, signifying the importance of research in this area [32, 33].

Due to the mental health implications highlighted in previous research, it is crucial to further understand the feelings and experiences of clinically referred gender-diverse young people in a rapidly changing context. The present research utilises specially formulated questionnaires, which focus on key clinical access points to gender services. The first of these questionnaires centres on pre-service experience, specifically on the experience in the six months before attending gender services. In this way, findings can be considered as a ‘baseline’, and in relation to previous literature, as well as in comparison to their subsequent experiences during access to gender services.

Understanding how young people navigate and experience their gender identity in the context prior to clinical support in services allows for a greater understanding of support needs and where clinical treatment may need to be focused to improve the mental health outcomes of gender-diverse young people.

Therefore, the present research aims to provide a demographic overview of clinically referred gender-diverse young people before service attendance to understand self-reported aspects of their gender identity and how they feel about them, including:

1. Social transition status, transphobic bullying, social connections, distress, and feelings about the body;
2. How they perceive others’ acceptance of their gender identity prior to clinical input.

## Methods

### Design

A retrospective observational analysis including young people and caregivers attending the Gender Identity Development Service (GIDS) in the British Isles was carried out. Young people and caregivers that completed the baseline measure of the GIDS Gender Questionnaire (GIDS-GQ) between April 2021 and February 2022 were included. Analysis was carried out from March 2022 to July 2022. Due to the retrospective nature of this review and use of full anonymisation, exemption for ethics was granted.

### Materials

The GIDS-GQ covers a wide range of gender-specific experiences unique to clinically referred gender-diverse young people and was developed in consultation with clinicians, stakeholders, and families attending the service. It is comprised of four separate questionnaires dependent on the age of the individual (caregiver completion for under 12-year-olds and service user completion for 12 + year olds) and stage of treatment (before attending the service (baseline) or during service attendance). Stakeholders and their families were consulted during the creation of the GIDS-GQ and felt that some concepts may be more difficult to grasp for those under age 12. Considerations also needed to be made around the reading age of young people attending services. Additionally as the questionnaire is online, and further clarifying questions could not be asked during completion, it was felt that caregiver completion for under 12-year-olds was most appropriate.

The baseline questionnaires focus on young people’s experiences in the six months prior to attending the service, whilst during service questionnaires assess their time in the GIDS. The questionnaire is composed of 38–60 items for young people over 12 (12+) and 24–38 items for caregivers of young people under 12 (U12).

Items related to:

- young people’s gender identity and related gender distress,
- social transition,
- past (lifetime) and present (in the last six months) experiences with transphobic bullying and treatment from others,
- their relationship with their body (only included for young people 12+), and.
- their changes in mood and connections with others.

Item responses include a combination of Likert responses [degrees of emotional responses (5 and 3 point) / Frequency (5 point)], Yes/Sometimes/No responses, numerical responses (age), and qualitative responses.

All distress data is reported using a 5-point Likert Scale (0 = Not at all distressed, 1 = Slightly distressed, 2 = Somewhat distressed, 3 = Moderately distressed, 4 = Extremely distressed) and all happiness scores using a separate 5-point Likert Scale (0 = Extremely happy, 1 = Somewhat happy, 2 = Neither happy nor unhappy, 3 = Somewhat unhappy, 4 = Extremely unhappy). Items related to frequency are also reported using a 5-point Likert scale (1 = Always, 2 = Often, 3 = Sometimes, 4 = Rarely, 5 = Never).

Social transition status is reported according to a combination of items. A full social transition in under 12s is defined as having changed their name informally or by deed poll, having asked others to use pronouns that reflect their gender identity, and having their caregivers define their gender identity as fixed and different to their gender assigned at birth (this included identifying as non-binary). As those 12 + years completed their own questionnaires, additional items included: having permanently changed their appearance in accordance with their current gender identification with family, friends, at school, with strangers, and online. Parental reports querying gender fluidity are also not asked in the 12 + questionnaires. Young people that report some but not all of these aspects of social transition are categorised as partially socially transitioned.

### Participants

Data were collected from young people and caregivers attending the GIDS in the British Isles. The GIDS-GQ is shared with all young people (or caregivers for those aged under 12) attending the service. Participants were eligible for the current study if they had completed the baseline measure of the GIDS-GQ between April 2021 and February 2022. Ethnicity and date of birth data were taken from an online patient record system.

### Procedure

Young people (12 + years old) or caregivers (where young people were under 12 years old) completed the baseline measure of the GIDS-GQ after approximately their first appointment with the Service. Questionnaires were emailed to participants via an online survey (Qualtrics™).

### Analysis

Most analyses were descriptive and focused on the demography of the cohorts and their answers to the selected questions. Data are reported as means, standard deviations, percentages, and absolute values where relevant. All data reported from questionnaires completed by caregivers, including demographics, refers to the young people (U12) under their care.

In addition, an independent-samples t-test was conducted using IBM SPSS 25 to assess whether the age of the young people in the sample was representative of the whole cohort of young people accessing GIDS in the same timeframe.

Data were collected from individuals that completed the questionnaire between April 2021 and February 2022. Participants with 25% or more missing items were excluded from analysis. Data were extracted for analysis on: 15/02/2022.

## Results

### Participants

Eighty-four participants initially completed the questionnaire, with three participants excluded owing to missing data (75+% questionnaire unanswered). The final sample consisted of 81 young people (12+: 73, U12: 8), *M* = 15.77 (*SD* = 1.83, range = 9–17), who were predominantly assigned female at birth (89%) and of a white ethnicity (61.7%).

Assigned gender and age comparisons were explored between the present sample and the whole cohort of 790 GIDS young people who first attended the service in this timeframe to understand whether this sample was representative of the whole cohort. The assigned gender split of the study sample (88.9% assigned female at birth, 11.1% assigned male at birth) represented the whole cohort of young people (72.8% assigned female at birth, 27.2% assigned male at birth) well. However, age at first appointment significantly differed *t*(93.18) = -6.95, *p* < .001, 95% CI [-1.88, -1.05], with those completing the GIDS-GQ being younger (*M* = 15.77, *SD* = 1.83) than the whole cohort (*M* = 17.23, *SD* = 1.61). This is owing to young people over age 17 being less likely to attend the GIDS (and more often seeking a referral to adult gender services), thus baseline GIDS-GQs are shared less frequently with young people over 17 years old. A full breakdown of general characteristics of included participants is presented in Table 1.

### Experience of gender distress and social transition

Most young people (96.3%) reported that their gender identity was different to their gender assigned at birth (2.5% unsure, 1.2% did not answer) and all who answered had felt this way for more than 6 months. When asked about their experience of gender distress, 92.6% stated they felt some distress (from ‘extremely’ to ‘slightly’), and only 7.4% indicated no distress at all. Of the 7.4% who reported no distress, 83.3% were over 12 years old, and 50% reported being in puberty (16.7% not in puberty, 33.3% not reported).

All young people in the sample had undergone some aspect of social transition, with 75.3% of the sample (12+: 87%, U12: 13%) categorised as fully socially transitioned and the remaining 24.7% (12+: 100%) categorised as partially socially transitioned. Of the partially socially transitioned group, 90% stated that physical appearance was not consistent across contexts, with young people presenting as their gender assigned at birth 60% of the time with strangers, 45% of the time at school, 35% of the time with their family, 20% of the time with friends, and 15% of the time online. Some of these young people (30%) also stated that they had not consistently asked others to use their personal pronouns. Whilst a smaller sub-set (10%) stated that they had not changed their name informally or by deed poll.

### External negotiation of gender and bullying

Most young people (97.4%) reported having asked family and/or friends to use pronouns that reflect their gender identity (91.4% yes, 6% sometimes). Only 1.2% had not and 1.2% did not understand the question. Those that had asked others to use their preferred pronouns reported feeling either extremely happy, somewhat happy or neither happy nor unhappy when these were always (38%) or often (49.4%) endorsed. However, when preferred pronouns were only used sometimes (12.6%) participants reported a wider range of happiness scores, including somewhat unhappy.

Young people reported experiencing more transphobic bullying in the past (lifetime) (43.2% yes, 21% sometimes) than presently (last 6 months prior to attending services) (4.9% yes, 7.4% sometimes), while also reporting more related distress in the past (48.5% extremely or moderately distressed, 29.4% somewhat or slightly distressed, 22% not at all distressed) than presently (12% extremely or moderately distressed, 7.6% somewhat or slightly distressed, 80.3% not at all distressed). Similarly, participants reported having been treated in a way that did not reflect their gender identity more often in the past (66.6% yes, 18.5% sometimes) than presently (6.2% yes, 43.2% sometimes; further detail can be found in Table 2).

### Body

Self-report on feelings about the body is only presented for young people aged 12+. 94.5% of the sample acknowledged disliked body parts, which consisted of primary and secondary sex characteristics (breasts (80.8%), genitals (37%), and hips (31.5%)) which aligned with the demography of the cohort. Only 55% respondents reported liked body parts (most commonly hair (23.3%) hands (23.3%), and eyes (20.5%)).

### Mood and connections

Young people were directly asked whether different aspects of their mood and connections had decreased, stayed the same, or increased during the last six months before attending the service. The majority of the sample reported experiencing a decrease in their mood (61.25%) during the 6 months prior to attending the service. Young people most commonly reported that their engagement in social situations (50%), connecting with other people (47.09%), and feeling close to others (47.09%) had also decreased during this period. However, friendships, and school attendance had stayed the same for around half the sample (56.35% and 55% respectively). Table 3 shows young people’s changes in mood and connections during the 6 months prior to attending the service.

**Table 1:** Demographic characteristics of the sample, including assigned sex at birth, age, puberty, and ethnicity

Assigned Sex at Birth					
	Female (*n* = 72)		Male (*n* = 9)		Total
12 + years	68		5		73
U12 years*^1^	4		4		8
Age					
	Female		Male		Total
12 + years	*M* = 16.36 years (*SD* = 0.96)		*M* = 14.88 years (*SD* = 1.61)		*M* = 16.26 years (*SD* = 1.07)
U12 years	*M* = 11.28 years (*SD* = 1.26)		*M* = 11.28 years (*SD* = 0.62)		*M* = 11.28 years (*SD* = 0.92)
Puberty*^2^					
	Female		Male		
	12 + years	U12 years	12 + years	U12 years	Total
In puberty	48	3	1	-	52
Not in puberty	12	1	1	3	17
Did not report	8	-	3	1	12
Ethnicity					
White			61.7%		
Mixed			1.2%		
Not recorded			16.1%		
Refused to report			3.7%		
Unable to choose			12.3%		

*^1^ Data for young people under 12 (U12) was reported by their caregivers.

*^2^ Puberty was self-reported for all participants.

**Table 2:** Past and present gender-related experiences of non-acceptance, including related distress

N = 81				
	Past (lifetime)		Present (last 6 months)	
	n	Related distress	n	Related distress
Yes	54	Extremely distressed: 32 Moderately distressed: 20 Somewhat distressed: 2	5	Extremely distressed: 3 Moderately distressed: 1 Somewhat distressed: 1
Sometimes	15	Extremely distressed: 3 Moderately distressed: 5 Somewhat distressed: 6 Slightly distressed: 1	35	Extremely distressed: 12 Moderately distressed: 12 Somewhat distressed: 7 Slightly distressed: 4
No	5*^1^	Somewhat distressed: 1 Not at all distressed: 1	36 *^2^	Somewhat distressed: 1 Slightly distressed: 3 Not at all distressed: 26
I do not want to answer this question	1	N/A	1	N/A
Blank	6	N/A	4	N/A

*1: Due to missing data, distress data is reported for only for 2 participants.

*2: Due to missing data, distress data is reported for only 30 participants.

**Table 3:** Gender-related changes in areas of mood and social connectedness in the past 6 months

N = 51 “In the last six months before attending the Service, did the way you feel/your child feels about your/their gender assumed at birth have an impact on any of the following (please select N/A if not applicable):”				
	Increased or Improved (n)	Stayed the Same (n)	Decreased or Deteriorated (n)	N/A (n)
My mood had:	4	12	34	1
Engaging in social situations and/or social interactions had:	4	15	29	3
Connecting with other people had:	4	22	24	1
Feeling close to other people had:	3	21	24	3
My friendships had:	6	28	16	1
My attendance at school or college had	1	34	12	3

## Discussion

The present study is the first to utilise the GIDS-GQ to provide an overview of the feelings and experiences of gender-diverse young people prior to commencing gender care in a specialist service, with the primary aim of understanding each young person’s experience to help inform where clinical treatment may need to be focused. Overall, within this sample, the majority of young people had undergone a full social transition, and had more often experienced transphobic bullying and not being treated in a way that reflected their gender identity in the past than presently. They also described disliked body parts more readily than liked body parts, and had most commonly experienced a decrease in mood and social connections.

Most young people (75.3%) had fully socially transitioned before they were first seen at the GIDS, as has been reported by others [25]. This had increased from previous reportage by this service [19] by 20.7%. Due to an increase in referrals in recent years [6–8], there has been a consequential increase in the waiting period before accessing services, and many young people are now first seen at an older age compared to previous years. As a result, a growing number of young people may have chosen to fully socially transition during the wait for services, as this is often one of the first and most meaningful steps taken by a gender-diverse person to live as their identified gender [22]. For those who had partially socially transitioned, the majority presented as their identified gender least frequently with strangers (40% of the time). This may be owing to concerns around experiencing discrimination from strangers as is commonly reported by gender-diverse communities [34], resulting in feeling uncomfortable presenting as their identified gender in these settings. Instead, young people were most comfortable presenting as their identified gender online, where they may have access to unique communities for gender-diverse adolescents to express and understand their gender identity, as has been found elsewhere [35, 36].

Of interest, more young people reported experiencing the external/distal stressors of transphobic bullying (64.2%) and non-acceptance (85.1%) in the past (lifetime) than presently (last 6 months prior to service attendance) (transphobic bullying: 12.3%; non-acceptance: 49.4%). This may be owing to the sample being further along in their social transition at the time of answering the questionnaire than in the past, resulting in a reduction of misgendering and more acceptance from others. Indeed, Kattari et al. [37] reported that adult gender-diverse samples who reported a higher level of ‘passing’ as their identified gender were also less likely to report discrimination. In addition, fewer instances of these external/distal stressors may be due to increased visibility and awareness of gender diversity in today’s society compared to ‘the past’, including in school settings [38, 39]. Levels of reported distress experienced in the past owing to transphobic bullying were also high in the present sample, indicating that these external/distal stressors were likely contributing negatively to mental health as has been described elsewhere [15–17]. Considering acceptance of gender identity, young people placed much importance on pronoun use, with 97.4% of the sample asking others to use preferred pronouns and expressing more satisfaction when pronouns were endorsed. Indeed, Brown et al. [40] found that others’ endorsement of preferred pronouns contributed to gender-diverse young people feeling supported and validated in their identified gender, and reduced reported emotional distress.

Most young people stated that they felt distress related to their assigned gender at birth, as would be expected in clinically referred young people [5]. Of note, most young people (94.5%) reported disliking parts of their body, with the most commonly reported (breasts, genitals, and hips) reflecting the demographic of the cohort (assigned female at birth young people in puberty), and the escalating distress that can be associated with the development of primary and secondary sex characteristic during puberty [9–11].

Importantly, many young people reported still experiencing the external/distal stressors highlighted by the Gender Minority Stress Framework of transphobic bullying (12.3%) and/or non-acceptance (49.4%), as well as reporting related distress [12–14]. In addition, the present sample most commonly reported that their experience of gender-related distress directly resulted in a decrease in their mood and all areas of social connectedness except friendships and school attendance, which had ‘stayed the same’. These findings are crucial as relationships with caregivers, peers, and community connectedness are found to be central to the psychological wellbeing of gender-diverse young people [41], and the Minority Stress Model highlights social connectedness as a key protective factor against minority stressors, such as bullying and non-acceptance [12–14]. Indeed, negative peer relations have been noted as instrumental in predicting behavioural and emotional difficulties in clinically referred young people [32, 33].

### Limitations

The sample consisted of a predominantly adolescent (pubertal) group and the majority were assigned female at birth, which limits study generalisability and comparison. However, the demographics of the sample does reflect the higher proportion of pubertal assigned female at birth young people referred to gender services, both in the GIDS [6, 19, 33], and elsewhere [7, 8, 42], although more recent assessment of community samples indicate a more even ratio in the United States [43].

Of note, the sample was significantly younger than the whole cohort of young people who first accessed GIDS in the timeframe. This may be owing to young people aged over 17 years old being more likely to be directly referred onto adult gender services. As it would be unethical to collect data from young people not requiring the service, this age group may receive questionnaires less frequently and not be represented in the study sample.

The majority of the sample (61%) were noted as a white ethnicity, which mirrors GIDS referral demographics [6, 44] and indicates that these findings may not be representative of ethnic minority gender-diverse youth, who are underrepresented in this research and across gender services more generally.

It is important to note that the baseline GIDS-GQ is completed after attending up to three appointments in the service, and, despite the questionnaire specifically focussing on experiences prior to service access, these initial appointments may have influenced questionnaire response. Indeed, it is important to note that a sub-set of young people (7.4%) reported no gender-related distress, despite this being a key referral acceptance criteria and an intrinsic part of assessment. It may be that these young people did not consider the six months prior to service attendance and responded in relation to their current experience at time of questionnaire completion. As young people would have had up to two appointments with the service at this point, the psychosocial support received during this time may have begun to reduce distress.

Additionally, ‘stayed the same’ responses for social connectedness questions are difficult to quantify in isolation as it is not clear whether the response refers to negative or positive experience. These questions become more meaningful over time, at subsequent response points.

## Conclusions

The GIDS-GQ is a new questionnaire, focused on understanding the individual needs of clinically referred young people and their experience of their gender journey. The present research found most young people to experience distress related to their gender identity, which most commonly resulted in a decrease in their mood and social connections. Additionally, most young people had undergone a full social transition and reported disliking parts of their body, with physical appearance varying across contexts for most young people who had partially socially transitioned. Young people reported more instances of transphobic bullying and non-acceptance in the past than at present, although it is important to note that many young people reported still experiencing these external/distal stressors. As the present work collected baseline (pre-clinical service treatment) experiences, future research is vital to compare these baseline responses to responses during service attendance. This would allow for further understanding about the influence of clinical support in relation to how young people identify, present, how they perceive others treat them, and, most vitally, how they experience their mental health and connection to others. As the Minority Stress Model highlights social connectedness as a key protective factor against minority stressors [12–14], future research is needed to understand how clinical support can help reduce the impact of these external/distal minority stressors by promoting social connectedness, incorporating such learnings into clinical practice and subsequent policy in clinical work with gender-diverse young people. Such work can allow for more consideration around tailored care pathways for gender-diverse youth, and further understanding of the differences and similarities in the experiences of young people attending the service. Furthermore, sharing the GIDS-GQ questionnaire with additional gender identity services would allow for further testing, to understand its broader utility.

## Data Availability

No data are available. Owing to the sensitive nature of this patient data, and to ensure anonymity, data are not available to share.

## Abbreviations

GIDS: Gender Identity Development Service
GIDS-GQ: Gender Identity Development Service - Gender Questionnaire
PRS: Peer Relation Scale
